# Supplementary material for: Machine Learning for Brain MRI Data Harmonisation: A Systematic Review
Source: Bioengineering (Basel). 2023 Mar 23;10(4):397. doi: 10.3390/bioengineering10040397 (PMC10135601; doi:10.3390/bioengineering10040397)
Supplement: Supplementary file 1 [file bioengineering-10-00397-s001.zip › bioengineering-2259748-supplementary.pdf]

Table S1. Papers used explicit approach

Paper	Dataset and # of subjects	Specific Model	Harmonized Domain	Evaluation	Training data	Validation Strategies	Library	Summary
Garcia-Dias et al., 2020 [1]	15,026 (public)	Random forest regression	volumes (T1-w)	Group-wise: P-values	1D vector of 101 features	Leave-one-scanner-out CV	Sklearn	A nonlinear regression model is trained, where the ground truth harmonized measures were estimated by ComBat harmonization methods [Codes are available].
Dewey et al., 2019 [2]	12 (institutional, travelling)	CNN	intensity (T1-w, T2-w, PD, FLAIR)	Subject-wise: SSIM, AE, DSC, PVD and volume bias<br>Group-wise: p-value from t-test	2D patches (128×128)	six-fold CV	Keras and Tensorflow	Model was trained using multi-contrast images to achieve site-to-site mapping. Final volumes are predicted in a 2.5D inference. The proposed method outperformed DL based latent space method and random forest-based method.
Tong et al., 2020 [3]	5 (travelling, institutional)<br>14 (travelling, public)	CNN	DKI measures: (DTI)	Subject-wise: MSE<br>Group-wise: Coefficient of Variation	3D patches (3×3×3)	Leave-p-subjects-out CV<br>Leave-one-tissue-out CV	-	Train a CNN model with the DTI images from one scanner while eight DKI measures estimated from DTI images obtained from the reference scanner. The proposed model outperformed non-ML algorithms.
Koppers et al., 2019 [4]	10 (travelling, public)	CNN	SH: (dMRI)	Subject-wise: MSE	3D patches (3×3×3)	Ten-fold CV (8:2)	-	Residual network was trained to learnt how to predict the SH coefficient and performance the mapping across two domains using the predicted SH features.

St-Jean et al., 2020 [5]	14 (travelling, public)	SDL	Raw signals (dMRI)	Subject-wise: MNE, error and % of difference<br>Group-wise: Hedges'g for t-test, KL divergence	3D patches (3 × 3 × 3)	three-fold CV, AIC minimisation	Sklearn	Apply mapping between scanners for matched acquisition protocols and then map the original and altered data sets toward a common harmonisation space which is created by randomly sampling data sets from all scanners [codes are available]
Zhao et al., 2019 [6]	263 (public)	GAN	cortical thickness maps (T1-w and T2-w)	Subject-wise: MAE, PSNR, Euclidean distances between any two scans<br>Group-wise: PCA, Cohen's d	2D cortical thickness map	Split dataset	-	GANs was trained to learn a site-to-site mapping. The proposed method was validated on both synthetic paired data and real unpaired data, where synthetic paired dataset was created by resampling.
Ren et al., 2021 [7]	246 (public)	GAN	intensity (T1-w, FLAIR)	Downstream task: Segmentation accuracy	2D patches (256 × 256)	leave-one-subject-out CV	PyTorch	By incorporating segmentation networks, GAN was trained and encouraged to produce the same segmentation via a segmentation-objective. The proposed method was compared and outperformed GAN-based methods [codes are available]
Robinson et al., 2020 [8]	1254 (public)	Transformer network	intensity (T1-w)	Downstream tasks: sex classification, age prediction	3D patches (64 × 64 × 64)	Split dataset	PyTorch	An image-and-spatial transformer network were trained to achieve site-to-site mapping. The proposed method was outperformed the GAN method. [codes are available]

Dewey et al., 2020 [9]	140 (institutional) 10 (travelling, institutional)	Auto-encoder	intensity (T1-w, T2-w)	Subject-wise: SSIM and PSNR	2D patches (224 × 192)	Split dataset	-	An auto-encoder is trained using paired T1-w and T2-w images from same subjects. Model's parameters were searched using grid search.
Zuo et al., 2021 [10]	120 (institutional) 7 (travelling, institutional)	Auto-encoder	intensity (T1-w, T2-w)	Subject-wise: SSIM and PSNR	2D patches (224 × 192)	Split dataset	PyTorch	Paired T1-w and T2-w from multiple sites were used to train the model. The proposed method outperform the histogram matching, GANs and the method presented in [9].
Zuo et al., 2021 [11]	20 (travelling, institutional) 100 (public)	Auto-encoder	intensity (T1-w, T2-w)	Subject-wise: SSIM, PSNR, DSC and PVD	2D patches (224 × 224)	Split dataset	PyTorch	The model was developed based on [9], [10]. The author proposed a 2.5D inference to produce the final volume. [codes are available]
Gao et al., 2019 [12]	489 (institutional) 10 (travelling, institutional)	GAN	Image-level features: intensity of T2-FLAIR	Subject-wise: PSNR, histogram correlation, SSIM, MSD, MSE and average disparity Downstream tasks: classification of diagnosis	2D slices (256 × 256)	Split dataset	-	Multiple generators and discriminators were used for each sites or site combinations to achieve the many-to-one mapping. The proposed method outperformed the histogram matching.
Liu et al., 2021 [13]	1 (travelling, public) 718 (public)	GAN	intensity (T1-w)	Subject-wise: DSC, SSIM, Euclidean distances between any two scans	2D slices (128 × 128)	Split dataset	-	Mapping across domains utilises a style-encoder to learn the scanner-invariant style code in images.

Weninger et al., 2022 [14]	161 (travelling, public)	GAN	SH (dMRI)	Subject-wise: MSE	3D patches (40 × 40 × 40)	Split dataset	PyTorch	Travelling subject dataset which contains scans from 3T and 7T scanner were used. The results of supervised, unsupervised, and mixed training were compared.
Torbati et al., 2021 [15]	18 (travelling, institutional)	Auto-encoder	intensity (T1-w)	Subject-wise: SSIM, SD, MSE, MAD	2D slices	Train and test on the same dataset	Tensorflow and keras	The model was trained to first learn the embeddings with structural information and then harmonise based on the embeddings. The proposed method was outperformed the statistical methods [codes are available]
Tian et al., 2022 [16]	9 (travelling, public)	Auto-encoder	GM volume maps (T1-w)	Group-wise: p-value for ANOVA and spearman's correlation, KL divergence	2D slices (176×208)	Leave-one-subject-out CV	Tensorflow and keras	The autoencoder was trained and evaluated with traveling subject datasets. 2.5D interface was developed. The proposed model outperformed statistical harmonization methods [codes are available].
Fatania et al., 2022 [17]	310 (public)	Auto-encoder	Intensities (T1-w)	Group-wise: p-value for KS and t-test	2D slices	Split dataset	TensorFlow	The proposed model consists of an auto-encoder based on site-to-site translation. The experiment was tested on data from unseen

								scanners and outperformed statistical methods.
Arai et al., 2021 [18]	616 (public)	GAN	Intensities (T1-w)	Subject-wise: PSNR, MSE, SSIM<br><br>Group-wise: t-SNE for cluster visualisation<br><br>Downstream tasks: classification	3D volume (160 × 160 × 192)	5-fold CV	-	The proposed method used GAN model to achieve one-to-one mapping, aim with obtaining a scanner independent low-dimensional representation while preserving disease-related anatomical features.
Zhong et al., 2020 [19]	84 (institutional)	GAN	DTI-derived metric map (dMRI)	Subject-wise: AE, MSE<br><br>Group-wise: Cohen's d, p-value for U test, Pearson correlation	3D patches (30 × 30 × 8)	Six-fold CV	-	Dual GANs was trained to learn the mappings of DTI-derived metrics of age-matched neonates from two sites. The proposed approach outperformed the statistics method.
Moyer et al., 2020 [20]	15 (travelling; public)	Auto-encoder	SH (dMRI)	Subject wise: MSE<br><br>Group-wise: APE and Coefficient of Variation	1D vector of DWI signal	Split dataset	-	All scans were masked for WM tissue before converting them to the SH representation. Results for single and multiple sites mappings were compared and results showed that they outperformed to the method presented in [21].

Table S2. Papers used implicit approach

Paper	Dataset	Specific Techniques	Modality	Evaluation	Training input	Validation Strategies	Software	Summary
Guan et al., 2021 [22]	2572 (public)	Adversarial transfer learning	T1-w: intensities	Group-wise: visualization using t-SNE<br>Classification (downstream tasks)	3D, raw intensities	5-fold CV	PyTorch	The proposed DA technique uses an attention discovery module to locate disease-related regions in brain MRIs.
Dinsdale et al., 2021 [23]	8417 (public)	Adversarial transfer learning	T1-w: intensities	Group-wise: visualization using t-SNE<br>Downstream tasks: Segmentation, Brain age regression	2D patches of size 128 × 128	5-fold CV	PyTorch	Adapt an iterative update approach to the adversarial learning [codes are available].
Orbes-Arteaga et al., 2019 [24]	133 (public)	Adversarial transfer learning	FLAIR: intensities	Downstream tasks: Segmentation	2D slices of size 256 × 256	Split dataset	-	The proposed method combines data augmentation strategy and adversarial networks to improve the main task performance.
Ackaouy et al., 2020 [25]	53 (public)	Adversarial transfer learning	FLAIR: intensities	Downstream tasks: Segmentation	3D patches of size 16 × 16 × 16	Split dataset	Keras	The proposed method is based on the Optimal Transport, which learns a shared embedding for the source and target domains while preserving the discriminative information used by the discriminator.

Zhang et al., 2019 [26]	1506 (public)	Adversarial transfer learning	T1-w: intensities	Downstream tasks: Classification	2D slices of size 256 × 256	2-fold CV	PyTorch	The model utilizes a cycle feature adaptation module to harmonize features.
Yousefnezhad et al., 2020 [27]	142 (public)	Feature extraction	fMRI: time-series raw signals	Downstream tasks: Classification	2D time-series raw signals (1D flatten voxel)	Leave-one-subject out CV	PyTorch	First extracts a set of common features for all subjects in each site, and then then maps these site-specific features to a global shared space.
Wang et al., 2020 [28]	468 (public)	Feature extraction	fMRI: functional connectivity	Downstream tasks: Classification	2D functional connectivity matrix of size 116 × 116	5-fold CV	-	The proposed method treats one site as a target domain and the remaining sites as source domains. Transform the source domains to a common space using low-rank representation.
Wang et al., 2022 [29]	609 (public)	Feature extraction	fMRI: functional connectivity	Downstream tasks: Classification	2D functional connectivity matrix of size 64 × 64	leave-one-out CV	-	Use a nested singular value decomposition method to mitigate inter-site heterogeneity and extract features by learning both local cluster-shared features across sites and global category-shared features.
Delisle et al., 2021 [30]	1118 (pubic)	Adversarial transfer learning	T1-w, T2: intensities	Downstream tasks: Segmentation	3D patches of size 32 × 32 × 32	Split dataset	PyTorch	The overall architecture consists of a generator, a segmenter that outputs a segmentation map from output of the generator and a discriminator [codes are available].

Huang et al., 2020 [31]	973 (public)	Adversarial transfer learning	fMRI: connectome-based features	Downstream tasks: Classification	1D vector of connectome-based features	10-fold CV	-	The classification network with adversarial module was trained on source domains and then tested on another unlabelled target domain.
C. Monte-Rubio et al. [32]	303 (institutional)	Fine-tuning	T1-w: segmentation map	Downstream tasks: Classification	2D voxel wise segmented data of GM and WM	Leave-one-out-out CV	R package	Train a Gaussian process site classifier to estimate the harmonisation parameters. Then encode the parameters for the main classifier.
Chen et al., 2020 [33]	1380 (public)	Fine-tuning	Diffusion MRI: tract-specific analysis results	Downstream tasks: Brain age regression	3D image-feature data with size of 100 × 76 × 9	10-fold CV	MATLAB	Use model constructed with a large dMRI dataset as the source domain, and then transfer it to three target domains with distinct acquisition scenarios [codes are available].
Wachinger and Reuter, 2016 [34]	1350 (public)	Fine-tuning	T1-w: image-features	Downstream tasks: Classification	1D vector of cortical thickness, brain volume and shape features	Split dataset	R package	The DA technique based on the fine-tuning via instance weighting was used to transfer between the classifier on two databases.
Ghafoorian et al., 2017 [35]	439 (public)	Fine-tuning	T1-w, FLAIR: intensities	Downstream tasks: Segmentation	2D patches of size 32 × 32	Split dataset	-	The DA technique based on fine-tuning was used to transfer the original to the follow-up scan domain due to a change of scanner parameters. The same segmentation parameters. The same segmentation annotations were used for both domain during training and testing.
Balboni et al., 2022 [36]	73 (institutional)	Fine-tuning	T1-w: intensities	Downstream tasks: Segmentation	3D patches of size of 32 × 64 × 64	Split dataset	Tensorflo w and keras	This study fine-tunes the network trained in a previous study using ADNI datasets to segment scans in

								three new datasets [codes are available].
Shi et al., 2021 [37]	343 (public)	Fine-tuning	fMRI: functional connectivity	Downstream tasks: Classification	2D functional connectivity matrix of size 90 × 90	Five-fold CV	-	The DA method is based on a three-way decision model based on triangular fuzzy similarity and divide the objects in the target domain with coarse granularity.
van Opbroek et al., 2015 [38]	56 (public)	Fine-tuning	T1, T2, and FLAIR: intensities	Downstream tasks: Segmentation	2D voxel-wise data	CV	-	Perform regions and lesion segmentation using transfer learning considering unbalance training data.
Wang et al., 2022 [39]	2641 (public)	Fine-tuning	T1-w: intensities	Group-wise: visualization using t-SNE<br>Downstream tasks: Classification	3D volume of size 193 × 229 × 193	5-fold CV	PyTorch	Introduce a regularization term in fine-tuning process of domain adaptation for diverse population and imaging devices.
Shi et al., 2022 [40]	452 (public)	Fine-tuning	fMRI: functional connectivity	Downstream tasks: Classification	1D voxel wise vector	Split dataset	-	Estimate the weight coefficient of each source domain to accurately describe the importance. And then adjust the sampling weights for the main network, using optimal transport theory.
Opbroek et al., 2015 [41]	61 (public)	Fine-tuning	T1-w: intensity	Downstream tasks: Segmentation	2D images	Leave-one-domain-out/ Leave-one-subject-out CV	LIBSVM	Estimate a weight to each image based on the distribution of its voxels in the feature space. The voxels and weights of the training images are then used to train a weighted classifier.

Ma et al., 2018 [42]	300 (public)	Multi-task learning	T1-w: intensities	downstream tasks : Classification	1D voxel-wise data	5-fold CV	-	The proposed method performed multi-task learning for multi-site data learning.

1D: one dimensional

2.5D: pseudo three dimensional

2D: two dimensional

3D: three dimensional

ADL: adaptive dictionary learning

AE: Absolute error

AIC: Akaike information criterion

APE: Absolute Percentage Error

CNN: Convolutional neural network

CV: cross validation

DA: domain adaptation

DKI: diffusion kurtosis imaging

DSC: Dice similarity coefficient

GAN: Generative Adversarial Networks

KL: Kullback–Leibler

KS: Kolmogorov–Smirnov

MAD: Mean absolute deviation

MAE: mean solute error

MD: mean diffusivity

MNE: mean normalized error

MSD: Mean squared deviation

MSE: root mean square error

PSNR: peak-signal-to-noise ratio

PVD: percent volume difference

ROI: region of interest

SDL: spare dictionary learning

SH: spherical harmonic

SSIM: structural similarity index measure

Table S3: Quality Assessment of All Included Articles

	Q1	Q2	Q3	Q4	Q5	Q6	Q7	Q8	Q9	Q10	Score
[11]	1	1	1	1	1	1	1	1	1	1	10
[22]	1	1	1	1	1	1	1	1	1	1	10
[42]	1	1	1	1	1	1	1	1	1	0.5	9.5
[33]	1	1	1	1	1	1	1	1	0	1	9
[2]	1	1	1	1	1	1	1	0	1	1	9
[10]	1	1	1	1	1	0.5	1	1	1	0.5	9
[16]	1	1	1	1	1	1	1	1	0	1	9
[20]	1	1	1	1	1	1	1	0	1	1	9
[28]	1	1	1	1	1	1	1	1	1	1	9
[29]	1	1	1	1	1	1	1	1	1	1	9
[30]	1	1	1	1	1	1	1	1	0	1	9
[32]	1	1	1	1	1	1	1	1	0	1	9
[39]	1	1	1	1	1	1	1	0	1	1	9
[40]	1	1	1	1	1	0.5	1	1	1	0.5	9
[5]	1	1	1	1	1	1	0.5	0	1	1	8.5
[7]	1	1	1	0.5	1	1	1	0	1	1	8.5
[12]	1	1	1	1	1	1	1	1	0	0.5	8.5
[34]	1	1	1	1	1	1	1	1	0	0.5	8.5
[38]	1	1	0	1	1	1	1	1	0	1	8
[1]	1	1	1	1	1	1	1	0	0	1	8
[15]	1	1	1	1	1	1	1	1	0	0.5	8
[18]	1	1	1	1	1	1	1	0	0	1	8
[19]	1	1	1	0.5	1	1	0.5	1	0	1	8
[35]	1	1	1	1	1	1	1	0	0	1	8
[23]	1	1	0.5	1	1	1	1	0	0	1	7.5
[3]	1	1	1	0	1	0.5	1	1	0	1	7.5
[13]	1	1	1	1	1	1	1	0	0	0.5	7.5
[17]	1	1	0.5	0.5	1	1	0.5	1	0	1	7.5
[24]	1	1	0.5	1	0.5	1	0.5	1	1	0	7.5
[26]	1	1	1	1	1	0.5	0.5	0	1	0.5	7.5
[27]	1	1	1	0.5	0.5	0.5	1	1	1	0	7.5
[31]	1	1	1	1	0.5	0.5	0.5	1	1	0	7.5
[37]	1	1	1	0.5	0.5	1	1	0	1	0.5	7.5
[4]	0.5	1	1	1	1	1	0.5	0	1	0	7
[25]	1	1	1	1	1	1	0.5	0	0	0.5	7

[36]	1	1	1	1	1	1	0.5	0	0	0.5	7
[41]	1	1	1	0.5	1	0.5	1	0	0	1	7
[14]	1	0.5	1	1	1	1	0.5	0	0	0.5	6.5
[8]	1	1	0.5	0.5	1	0.5	0.5	0	1	0	6
[6]	0.5	1	0.5	0.5	1	1	1	0	0	0	5.5
Average	0.98	0.99	0.91	0.85	0.95	0.90	0.86	0.53	0.475	0.70	8.16

Sub-Table of S3 (S3-1): Quality assessment of all articles that used explicit approaches

	Q1	Q2	Q3	Q4	Q5	Q6	Q7	Q8	Q9	Q10	Score
[11]	1	1	1	1	1	1	1	1	1	1	10
[2]	1	1	1	1	1	1	1	0	1	1	9
[10]	1	1	1	1	1	0.5	1	1	1	0.5	9
[16]	1	1	1	1	1	1	1	1	0	1	9
[20]	1	1	1	1	1	1	1	0	1	1	9
[5]	1	1	1	1	1	1	0.5	0	1	1	8.5
[7]	1	1	1	0.5	1	1	1	0	1	1	8.5
[12]	1	1	1	1	1	1	1	1	0	0.5	8.5
[1]	1	1	1	1	1	1	1	0	0	1	8
[15]	1	1	1	1	1	1	1	1	0	0.5	8
[18]	1	1	1	1	1	1	1	0	0	1	8
[19]	1	1	1	0.5	1	1	0.5	1	0	1	8
[3]	1	1	1	0	1	0.5	1	1	0	1	7.5
[13]	1	1	1	1	1	1	1	0	0	0.5	7.5

[17]	1	1	0.5	0.5	1	1	0.5	1	0	1	7.5
[4]	0.5	1	1	1	1	1	0.5	0	1	0	7
[9]	1	1	1	0	1	1	1	0	0	0.5	6.5
[14]	1	0.5	1	1	1	1	0.5	0	0	0.5	6.5
[8]	1	1	0.5	0.5	1	0.5	0.5	0	1	0	6
[6]	0.5	1	0.5	0.5	1	1	1	0	0	0	5.5
Total	0.95	0.98	0.93	0.78	1.00	0.93	0.85	0.40	0.40	0.70	7.88

Sub-Table of S3 (S3-2): Quality assessment of all articles that used implicit approaches

	Q1	Q2	Q3	Q4	Q5	Q6	Q7	Q8	Q9	Q10	Score
[22]	1	1	1	1	1	1	1	1	1	1	10
[33]	1	1	1	1	1	1	1	1	0	1	9
[42]	1	1	1	1	1	1	1	1	1	0.5	9.5
[28]	1	1	1	1	1	1	1	1	1	1	9
[29]	1	1	1	1	1	1	1	1	1	1	9
[30]	1	1	1	1	1	1	1	1	0	1	9
[32]	1	1	1	1	1	1	1	1	0	1	9
[38]	1	1	0	1	1	1	1	1	0	1	8
[39]	1	1	1	1	1	1	1	0	1	1	9
[40]	1	1	1	1	1	0.5	1	1	1	0.5	9
[23]	1	1	0.5	1	1	1	1	0	0	1	7.5

[34]	1	1	1	1	1	1	1	1	0	0.5	8.5
[35]	1	1	1	1	1	1	1	0	0	1	8
[36]	1	1	1	1	1	1	0.5	0	0	0.5	7
[41]	1	1	1	0.5	1	0.5	1	0	0	1	7
[24]	1	1	0.5	1	0.5	1	0.5	1	1	0	7.5
[26]	1	1	1	1	1	0.5	0.5	0	1	0.5	7.5
[27]	1	1	1	0.5	0.5	0.5	1	1	1	0	7.5
[31]	1	1	1	1	0.5	0.5	0.5	1	1	0	7.5
[37]	1	1	1	0.5	0.5	1	1	0	1	0.5	7.5
[25]	1	1	1	1	1	1	0.5	0	0	0.5	7
Total	1.00	1.00	0.90	0.93	0.90	0.88	0.88	0.62	0.52	0.69	8.3

1 = “yes”, 0.5 = “partial”, 0 = “no”.

Q1: Are the research aims clearly defined?

Q2: Is the data collection procedure or the datasets clearly defined?

Q3: Is the data pre-processing procedure clearly defined?

Q4: Is the characteristics of the input data clearly described?

Q5: Are the ML techniques well defined?

Q6: Is the training procedure clearly defined?

Q7: Are the results and findings clearly stated?

Q8: Are there any comparative analyses (statistical vs ML)?

Q9: Are there any comparative analyses (ML vs ML)?

Q10: Are the limitations of the study specified?

References

[1] R. Garcia-Dias *et al.*, "Neuroharmony: A new tool for harmonizing volumetric MRI data from unseen scanners," *NeuroImage*, vol. 220, p. 117127, Oct. 2020, doi: 10.1016/j.neuroimage.2020.117127.

[2] B. E. Dewey *et al.*, "DeepHarmony: A deep learning approach to contrast harmonization across scanner changes," *Magn. Reson. Imaging*, vol. 64, pp. 160–170, Dec. 2019, doi: 10.1016/j.mri.2019.05.041.

[3] Q. Tong *et al.*, "A deep learning–based method for improving reliability of multicenter diffusion kurtosis imaging with varied acquisition protocols," *Magn. Reson. Imaging*, vol. 73, pp. 31–44, Nov. 2020, doi: 10.1016/j.mri.2020.08.001.

[4] S. Koppers, L. Bloy, J. I. Berman, C. M. W. Tax, J. C. Edgar, and D. Merhof, "Spherical Harmonic Residual Network for Diffusion Signal Harmonization," in *Computational Diffusion MRI*, Cham, 2019, pp. 173–182. doi: 10.1007/978-3-030-05831-9_14.

[5] S. St-Jean, M. A. Viergever, and A. Leemans, "Harmonization of diffusion MRI data sets with adaptive dictionary learning," *Hum. Brain Mapp.*, vol. 41, no. 16, pp. 4478–4499, 2020, doi: https://doi.org/10.1002/hbm.25117.

[6] F. Zhao *et al.*, "Harmonization of Infant Cortical Thickness Using Surface-to-Surface Cycle-Consistent Adversarial Networks," *Med. Image Comput. Comput.-Assist. Interv. MICCAI Int. Conf. Med. Image Comput. Comput.-Assist. Interv.*, vol. 11767, pp. 475–483, Oct. 2019, doi: 10.1007/978-3-030-32251-9_52.

[7] M. Ren, N. Dey, J. Fishbaugh, and G. Gerig, "Segmentation-Renormalized Deep Feature Modulation for Unpaired Image Harmonization," *IEEE Trans. Med. Imaging*, vol. 40, no. 6, pp. 1519–1530, Jun. 2021, doi: 10.1109/TMI.2021.3059726.

[8] R. Robinson *et al.*, "Image-Level Harmonization of Multi-site Data Using Image-and-Spatial Transformer Networks," in *Medical Image Computing and Computer Assisted Intervention – MICCAI 2020*, Cham, 2020, pp. 710–719. doi: 10.1007/978-3-030-59728-3_69.

[9] B. E. Dewey *et al.*, "A Disentangled Latent Space for Cross-Site MRI Harmonization," in *Medical Image Computing and Computer Assisted Intervention – MICCAI 2020*, Cham, 2020, pp. 720–729. doi: 10.1007/978-3-030-59728-3_70.

[10] L. Zuo *et al.*, "Information-Based Disentangled Representation Learning for Unsupervised MR Harmonization," in *Information Processing in Medical Imaging*, Cham, 2021, pp. 346–359. doi: 10.1007/978-3-030-78191-0_27.

[11] L. Zuo *et al.*, "Unsupervised MR harmonization by learning disentangled representations using information bottleneck theory," *NeuroImage*, vol. 243, p. 118569, Nov. 2021, doi: 10.1016/j.neuroimage.2021.118569.

[12] Y. Gao, Y. Liu, Y. Wang, Z. Shi, and J. Yu, "A Universal Intensity Standardization Method Based on a Many-to-One Weak-Paired Cycle Generative Adversarial Network for Magnetic Resonance Images," *IEEE Trans. Med. Imaging*, vol. 38, no. 9, pp. 2059–2069, Sep. 2019, doi: 10.1109/TMI.2019.2894692.

[13] M. Liu *et al.*, "Style Transfer Using Generative Adversarial Networks for Multi-site MRI Harmonization," in *Medical Image Computing and Computer Assisted Intervention – MICCAI 2021*, Cham, 2021, pp. 313–322. doi: 10.1007/978-3-030-87199-4_30.

[14] L. Weninger, M. Ahmad, and D. Merhof, "From Supervised to Unsupervised Harmonization of Diffusion Mri Acquisitions," in *2022 IEEE 19th International Symposium on Biomedical Imaging (ISBI)*, Mar. 2022, pp. 1–5. doi: 10.1109/ISBI52829.2022.9761445.

[15] M. E. Torbati, D. L. Tudorascu, D. S. Minhas, P. Maillard, C. S. DeCarli, and S. Jae Hwang, "Multi-scanner Harmonization of Paired Neuroimaging Data via Structure Preserving Embedding Learning," in *2021 IEEE/CVF International Conference on Computer Vision Workshops (ICCVW)*, Montreal, BC, Canada, Oct. 2021, pp. 3277–3286. doi: 10.1109/ICCVW54120.2021.00367.

[16] D. Tian *et al.*, "A deep learning-based multisite neuroimage harmonization framework established with a traveling-subject dataset," *NeuroImage*, p. 119297, May 2022, doi: 10.1016/j.neuroimage.2022.119297.

[17] K. Fatania *et al.*, "Harmonisation of scanner-dependent contrast variations in magnetic resonance imaging for radiation oncology, using style-blind auto-encoders," *Phys. Imaging Radiat. Oncol.*, vol. 22, pp. 115–122, Apr. 2022, doi: 10.1016/j.phro.2022.05.005.

[18] H. Arai, Y. Onga, K. Ikuta, Y. Chayama, H. Iyatomi, and K. Oishi, "Disease-Oriented Image Embedding With Pseudo-Scanner Standardization for Content-Based Image Retrieval on 3D Brain MRI," *IEEE Access*, vol. 9, pp. 165326–165340, 2021, doi: 10.1109/ACCESS.2021.3129105.

[19] J. Zhong *et al.*, "Inter-site harmonization based on dual generative adversarial networks for diffusion tensor imaging: application to neonatal white matter development," *Biomed. Eng. OnLine*, vol. 19, no. 1, p. 4, Jan. 2020, doi: 10.1186/s12938-020-0748-9.

[20] D. Moyer, G. Ver Steeg, C. M. W. Tax, and P. M. Thompson, "Scanner invariant representations for diffusion MRI harmonization," *Magn. Reson. Med.*, vol. 84, no. 4, pp. 2174–2189, 2020, doi: 10.1002/mrm.28243.

[21] H. Mirzaalian *et al.*, "Multi-site harmonization of diffusion MRI data in a registration framework," *Brain Imaging Behav.*, vol. 12, no. 1, pp. 284–295, Feb. 2018, doi: 10.1007/s11682-016-9670-y.

[22] H. Guan, Y. Liu, E. Yang, P.-T. Yap, D. Shen, and M. Liu, “Multi-site MRI harmonization via attention-guided deep domain adaptation for brain disorder identification,” *Med. Image Anal.*, vol. 71, p. 102076, Jul. 2021, doi: 10.1016/j.media.2021.102076.

[23] N. K. Dinsdale, M. Jenkinson, and A. I. L. Namburete, “Deep learning-based unlearning of dataset bias for MRI harmonisation and confound removal,” *NeuroImage*, vol. 228, p. 117689, Mar. 2021, doi: 10.1016/j.neuroimage.2020.117689.

[24] M. Orbes-Arteaga *et al.*, “Multi-domain Adaptation in Brain MRI Through Paired Consistency and Adversarial Learning,” *Domain Adapt. Represent. Transf. Med. Image Learn. Labels Imperfect Data First MICCAI Workshop DART 2019 First Int. Workshop MIL3ID 2019 Shenzhen Held Conjunction MICCAI 20*, vol. 2019, pp. 54–62, 2019, doi: 10.1007/978-3-030-33391-1_7.

[25] A. Ackaouy, N. Courty, E. Vallée, O. Commowick, C. Barillot, and F. Galassi, “Unsupervised Domain Adaptation With Optimal Transport in Multi-Site Segmentation of Multiple Sclerosis Lesions From MRI Data,” *Front. Comput. Neurosci.*, vol. 14, p. 19, Mar. 2020, doi: 10.3389/fncom.2020.00019.

[26] J. Zhang, M. Liu, Y. Pan, and D. Shen, “Unsupervised Conditional Consensus Adversarial Network for Brain Disease Identification with Structural MRI,” in *Machine Learning in Medical Imaging*, Cham, 2019, pp. 391–399. doi: 10.1007/978-3-030-32692-0_45.

[27] M. Yousefnezhad, A. Selvitella, D. Zhang, A. J. Greenshaw, and R. Greiner, “Shared space transfer learning for analyzing multi-site fMRI data,” in *Proceedings of the 34th International Conference on Neural Information Processing Systems*, Red Hook, NY, USA, Dec. 2020, pp. 15990–16000.

[28] M. Wang, D. Zhang, J. Huang, P.-T. Yap, D. Shen, and M. Liu, “Identifying Autism Spectrum Disorder With Multi-Site fMRI via Low-Rank Domain Adaptation,” *IEEE Trans. Med. Imaging*, vol. 39, no. 3, pp. 644–655, Mar. 2020, doi: 10.1109/TMI.2019.2933160.

[29] N. Wang, D. Yao, L. Ma, and M. Liu, “Multi-site clustering and nested feature extraction for identifying autism spectrum disorder with resting-state fMRI,” *Med. Image Anal.*, vol. 75, p. 102279, Jan. 2022, doi: 10.1016/j.media.2021.102279.

[30] P.-L. Delisle, B. Anctil-Robitaille, C. Desrosiers, and H. Lombaert, “Realistic image normalization for multi-Domain segmentation,” *Med. Image Anal.*, vol. 74, p. 102191, Dec. 2021, doi: 10.1016/j.media.2021.102191.

[31] Y.-L. Huang, W.-T. Hsieh, H.-C. Yang, and C.-C. Lee, “Conditional Domain Adversarial Transfer for Robust Cross-Site ADHD Classification Using Functional MRI,” in *ICASSP 2020 - 2020 IEEE International Conference on Acoustics, Speech and Signal Processing (ICASSP)*, May 2020, pp. 1190–1194. doi: 10.1109/ICASSP40776.2020.9054606.

[32] G. C Monte-Rubio *et al.*, “Parameters from site classification to harmonize MRI clinical studies: Application to a multi-site Parkinson’s disease dataset,” *Hum. Brain Mapp.*, Mar. 2022, doi: 10.1002/hbm.25838.

[33] C.-L. Chen *et al.*, “Generalization of diffusion magnetic resonance imaging–based brain age prediction model through transfer learning,” *NeuroImage*, vol. 217, p. 116831, Aug. 2020, doi: 10.1016/j.neuroimage.2020.116831.

[34] C. Wachinger and M. Reuter, “Domain Adaptation for Alzheimer’s Disease Diagnostics,” *NeuroImage*, vol. 139, pp. 470–479, Oct. 2016, doi: 10.1016/j.neuroimage.2016.05.053.

[35] M. Ghafoorian *et al.*, “Transfer Learning for Domain Adaptation in MRI: Application in Brain Lesion Segmentation,” in *Medical Image Computing and Computer Assisted Intervention − MICCAI 2017*, Cham, 2017, pp. 516–524. doi: 10.1007/978-3-319-66179-7_59.

[36] E. Balboni *et al.*, “The impact of transfer learning on 3D deep learning convolutional neural network segmentation of the hippocampus in mild cognitive impairment and Alzheimer disease subjects,” *Hum. Brain Mapp.*, Apr. 2022, doi: 10.1002/hbm.25858.

[37] C. Shi, X. Xin, and J. Zhang, “Domain Adaptation Using a Three-Way Decision Improves the Identification of Autism Patients from Multisite fMRI Data,” *Brain Sci.*, vol. 11, no. 5, Art. no. 5, May 2021, doi: 10.3390/brainsci11050603.

[38] A. van Opbroek, M. A. Ikram, M. W. Vernooij, and M. de Bruijne, “Transfer Learning Improves Supervised Image Segmentation Across Imaging Protocols,” *IEEE Trans. Med. Imaging*, vol. 34, no. 5, pp. 1018–1030, 2015, doi: 10.1109/TMI.2014.2366792.

[39] R. Wang, P. Chaudhari, and C. Davatzikos, “Embracing the disharmony in medical imaging: A Simple and effective framework for domain adaptation,” *Med. Image Anal.*, vol. 76, p. 102309, Feb. 2022, doi: 10.1016/j.media.2021.102309.

[40] C. Shi, X. Xin, and J. Zhang, “Domain adaptation based on rough adjoint inconsistency and optimal transport for identifying autistic patients,” *Comput. Methods Programs Biomed.*, vol. 215, p. 106615, Mar. 2022, doi: 10.1016/j.cmpb.2021.106615.

[41] A. van Opbroek, M. W. Vernooij, M. A. Ikram, and M. de Bruijne, “Weighting training images by maximizing distribution similarity for supervised segmentation across scanners,” *Med. Image Anal.*, vol. 24, no. 1, pp. 245–254, Aug. 2015, doi: 10.1016/j.media.2015.06.010.

[42] Q. Ma *et al.*, “Classification of multi-site MR images in the presence of heterogeneity using multi-task learning,” *NeuroImage Clin.*, vol. 19, pp. 476–486, Jan. 2018, doi: 10.1016/j.nicl.2018.04.037.
